# An observational simulation-based study of the accuracy of intercostal drain placement and factors influencing placement

**DOI:** 10.1016/j.afjem.2022.10.011

**Published:** 2022-11-16

**Authors:** Tessa Korda, Tammy Baillie-Stanton, Lara Nicole Goldstein

**Affiliations:** Division of Emergency Medicine, Faculty of Health Sciences, School of Clinical Medicine, University of the Witwatersrand, Johannesburg, South Africa

**Keywords:** Chest tubes, Thoracostomy, Clinical competence, Intercostal drain, Complications

## Abstract

**Introduction:**

South Africa's significant burden of trauma and respiratory disease requires the regular need for intercostal drain (ICD) insertion. ICD misplacement is associated with significant complications. The aim of this study was to assess ICD insertion site accuracy and the factors affecting accuracy by Emergency Department doctors.

**Methods:**

This was a prospective, observational simulation-based study. Prior to participant admission, pertinent thoracic structures were marked on two simulated patients using an invisible marker which could only be seen using ultraviolet light. One by one, study participants were then asked to place a sticker on each side of each patient's chest to indicate where they would insert an ICD. Sticker placements were photographed under ultraviolet light. Placement sites were compared according to the most appropriate and accurate position for insertion (within the area superior to the 6^th^ rib, posterior to pectoralis major and 1cm anterior to the mid-axillary line) as well as the British Thoracic Society's ‘triangle of safety’. Comparisons of accuracy were made between participant rank (junior vs senior doctor), participant experience, simulated patient BMI category and placement side.

**Results:**

Insertion site was accurate in 47% of placements and within the ‘triangle of safety’ in 51% of placements. Improved accuracy was associated with greater participant experience (61% vs. 37%, p<0.01), and left-sided chest placement (54% vs 40%, p = 0.02). No difference was found when comparing simulated patient BMI category (low vs high, 43% vs 51%, p=0.18) or participant rank (junior vs senior, 41% vs 51%, p=0.10).

**Conclusion:**

Overall accuracy of ICD insertion site was low, and appropriate accuracy was only associated with greater participant experience and left sided placement. Further studies to determine methods to improve ICD placement accuracy are needed.

## African relevance

■ High burden of respiratory disease and trauma in low- and middle-income countries results in a high rate of intercostal drain requirement.
■ Placement of intercostal drains is a critical skill in acute care medicine that is often carried out unsupervised by junior or inexperienced doctors working in overburdened public healthcare systems
■ Complications associated with misplaced intercostal drains are an unnecessary added strain on already overloaded healthcare systems.
■ Identification of factors associated with intercostal drain misplacement is an important step in the path to improving accuracy of placement and thus reducing the complications associated with misplacement.

## Background

Intercostal drain (ICD) insertion is a common Emergency Department (ED) procedure [1,2]. High rates of penetrating thoracic trauma and HIV-associated pulmonary illness have led to a significant number of patients presenting to the ED requiring ICD insertion in South Africa (SA) [3,4]. Training to insert an ICD correctly is an essential skill for doctors working in acute care [2,5].

Dufourq et al. found that resident doctors from a variety of disciplines perceived ICD insertion as the skill that they felt most competent in performing [6]. An SA study, however, identified procedure-related complications due to inadequate supervision of less experienced doctors inserting ICDs, a wide variability in the experience of staff regarding ICD insertion with higher complication rates occurring when ICD insertion was performed by staff outside of major trauma centres [2].

Complications of ICD insertion include but are not limited to extrathoracic placement, kinked tubing, subcutaneous or shallow insertion and inadequate fixation [7]. The classification of ICD complications is not standardised with complication rates varying widely between 1-40% [8]. Complications may result in longer hospital stays, additional investigations, and reparative surgery for visceral or vascular injury [9]. ICD complication-related morbidity and mortality, including financial implications is significant, particularly in a resource-constrained environment [2,8,9].

Several commonly used guidelines describe landmark-based palpation techniques for ICD placement including the British Thoracic Society (BTS), the Advanced Trauma Life Support (ATLS) and the European Trauma Course (ETC) methods [10], [11], [12]. The BTS guidelines refer to the ‘triangle of safety’ as the appropriate area for ICD insertion. This triangle constitutes: the lateral border of the pectoralis major anteriorly, the fifth intercostal space inferiorly and the lateral border of latissimus dorsi posteriorly, with the axilla as the apex [10]. This has been used as a reference to assess optimal ICD insertion previously [13]. ATLS guidelines recommend placement in the fourth or fifth intercostal space between the anterior and mid-axillary lines [11]. The Trauma Society of South Africa recommends the method taught by ATLS. The ETC guidelines recommend placement of an ICD one hand width below the patient's anterior axillary fold (armpit) just anterior to the mid-axillary line [12]. It has been shown that it is difficult to find the correct area by palpation alone [12,14]. A cadaveric study that assessed those three commonly used palpation techniques (BTS, ATLS, ETC) concluded that not all areas within the BTS ‘triangle of safety’ were, in fact, safe. Sites posterior to the line 1cm anterior to the mid-axillary line placed the long thoracic nerve at risk [12].

The aim of this study was to describe the accuracy of palpation-based ICD insertion site identification by ED staff of varying levels of experience and describe some of the factors associated with ICD misplacement.

## Methods

This was a prospective observational simulation-based study design. The study was approved by the University of the Witwatersrand Human Research Ethics Committee (M1911137). Written informed consent was obtained from all participants and simulated patients.

The study population comprised of ED doctors working in three academic hospitals in Johannesburg, SA. Doctors were classified as either junior or senior rank. Junior doctors included second year interns and community service medical officers i.e. Post-Graduate Year (PGY)-2 and PGY-3 doctors. Senior doctors included medical officers and emergency medicine residents (≥ PGY-4).

### Data collection

Data was collected from August – September 2020. Two male volunteers acted as simulated patients, one with a body mass index (BMI) of 20 and one with a BMI of 34. The simulated patients were positioned supine with arms behind their head and the head of the bed raised to 30^o^.

**Fig. 1:**
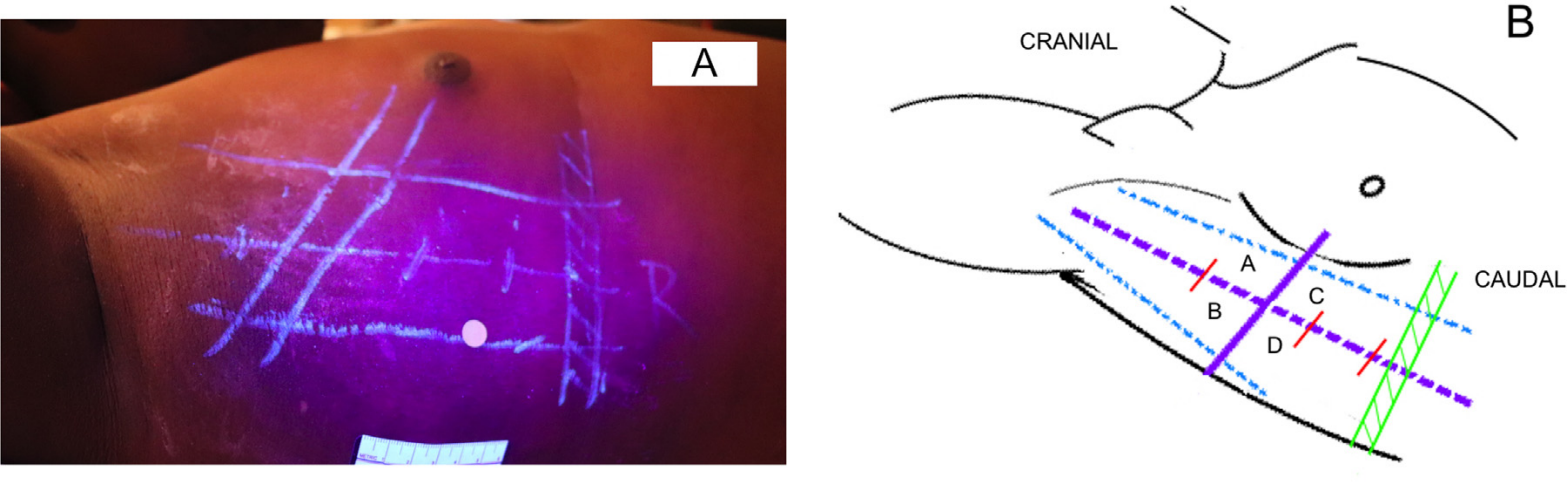
Photograph (A) and diagram (B) indicate how the right side of the thoracic wall was marked in the simulated patients with invisible ink (highlighted with ultraviolet light) prior to participant arrival. Photograph A additionally shows the circular sticker that was used by participants to indicate their preferred ICD insertion site. Diagram B has a green shaded area that represented the expiratory and inspiratory extents of the diaphragm. The anterior light blue line represented the lateral margin of pectoralis major and the posterior light blue line represented the lateral margin of latissimus dorsi. The dashed purple line represented the line 1cm anterior to mid-axillary line. The solid purple line represented the inferior margin of the 5^th^ intercostal space. The red lines that intersect the dashed purple line indicate the adjacent intercostal spaces’ inferior margins. Quadrants A-D shown as marked in diagram B.

Prior to the start of the data collection, ultrasound was used by the researcher (TK) to identify the relevant thoracic structures on the simulated patients (Fig. 1). The anatomy was then marked using an 8280 Special Securitas UV Marker (Edding, Germany) which has invisible ink which can only be seen if illuminated with ultraviolet light. The thoracic structures marked included the diaphragm at the end of inspiration and expiration, the 4^th^-7^th^ intercostal spaces, the lateral border of the pectoralis major muscle and the lateral border of the latissimus dorsi muscle (Fig. 1A). The chest wall was then divided into four quadrants (A-D) by a line drawn 1cm anterior to the mid-axillary line in a cranio-caudal direction, intersected by a second line along the inferior border of the fifth intercostal space (Fig. 1B). Quadrants A and B combined represented the BTS triangle of safety. For this study, Quadrant A represented the ‘accurate’ placement area, anterior to the line 1cm anterior to the mid-axillary line and superior to or within the 5^th^ intercostal space. Quadrants B-D represented areas of placement that were deemed not accurate as Quadrants B and C would be too posterior and Quadrants C and D would be too caudal on the thoracic wall. Although Quadrant B lies within the triangle of safety it is posterior to the line 1cm anterior to the mid-axillary line and so was not considered to be within the accurate area as per prior cadaveric findings [12].

Under normal ambient light, participants used their preferred palpation technique to locate and mark the ICD insertion site on each side of both of the simulated patients’ chest walls with a circular sticker. Participants were unable to see the markings made with the ultraviolet marker. To ensure consistency, participants were not allowed to change the position of the simulated patients. Each sticker was then photographed in situ using a Canon 850D SLR camera (Canon, SA), with the ultraviolet markings made visible using a Zartek ZA-490 UV Flashlight (Zartek, SA).

During analysis, marked structures were divided into two groups: muscular (latissimus dorsi and pectoralis major) and diaphragmatic structures (diaphragm/ subdiaphragmatic). Intercostal space placement was divided into 5^th^ intercostal space or superior and 6^th^ Intercostal space or inferior.

### Statistical analysis

Descriptive variables were presented as frequencies and percentages. The data was analysed using IBM SPSS Statistics, version 22 (2013). The Chi-square test was used to compare the accuracy of ICD placement between the following variables: rank, experience level, BMI, and side of placement. Significance testing was set at the 95% confidence level with a p-value less than 0.05 indicating statistical significance.

## Results

In total, 71 doctors participated, each marking 4 simulated ICD insertion sites, resulting in a total of 284 placements. The participant characteristics are shown in Table 1.

**Table 1:** Participant characteristics.

Ranking of doctor	Number of ICDs inserted previously	n (%)
**Junior doctors**		**27 (38)**
	< 30 ICDs	26 (96)
	> 30 ICDs	1 (4)
		
**Senior doctors**		**44 (62)**
	< 30 ICDs	15 (34)
	> 30 ICDs	29 (66)

ICD = intercostal drain.

### Accuracy of placement and placement within the triangle of safety

Of the 284 simulated ICDs, 47% were placed in the predefined ‘accurate’ area (Quadrant A) and 51% fell within the BTS triangle of safety (Quadrants A & B). The differences in placement between rank, experience level, simulated patient BMI and side of placement are shown in Table 2 for both the predefined accurate area and the BTS triangle of safety.

**Table 2:** Accuracy of placement and placement in BTS triangle of safety.

Accurate Placement (Quadrant A)			
	n (%)	n (%)	*p-value*
Rank	*Senior doctors*	*Junior doctors*	
	90 (51)*^#^*	44 (41)	*0.10*
Experience level	*>30 ICDs placed*	*<30 ICDs placed*	
	73 (61)	61 (37)	*< 0.01*
Simulated Patient BMI	*Low BMI*	*High BMI*	
	61 (43)	73 (51)	*0.18*
Side of placement	*Left*	*Right*	
	77 (54)	57 (40)	*0.02*
Placement in BTS Triangle of Safety (Quadrants A & B)			
	**n (%)**	**n (%)**	*p-value*
			
Rank	*Senior doctors*	*Junior doctors*	
	96 (55)	49 (45)	*0.10*
Experience level	*>30 ICDs placed*	*<30 ICDs placed*	
	77 (62)	68 (41)	*<0.01*
Simulated Patient BMI	*Low BMI*	*High BMI*	
	64 (45)	81 (57)	*0.04*
Side of placement	*Left*	*Right*	
	84 (59)	61 (43)	*0.01*

BTS = British Thoracic society; ICD = Intercostal drain; BMI = Body mass index

*Note: percentages reflect proportion of accurate and BTS placements within the indicated group – e.g. 51% of all placements by senior doctors were accurate^#^*

### Anatomical location of placements based on marked structures

Overall, 13% of placements were overlying marked structures: 4% overlying the diaphragmatic structures and 9% overlying the muscular structures. Senior doctors were significantly less likely to place the simulated ICDs over marked structures when compared to junior doctors (10% vs 19%, p = 0.03). Both senior doctors and junior doctors placed 4% of attempts over diaphragmatic structures. Senior doctors placed 6% over muscular structures, compared to 15% of junior doctors (p < 0.01). No significant differences were found regarding placements overlying marked structures when comparing experience level, simulated patient BMI and side of placement.

Participants placed 163 attempts (57%) within the 5^th^ intercostal space or superior. Almost a quarter of attempts (23%) were closest to the 7^th^ intercostal space. Participants with a higher experience level indicated a significantly larger proportion of simulated ICDs in the 5th intercostal space or superior (69% vs 49%, p < 0.01) whereas no significant difference was found when comparing rank, simulated patient BMI or side of placement.

## Discussion

Accurate placement of ICDs in patients is paramount in ensuring appropriate medical management and patient safety. It is thus vital that practitioners inserting ICDs are aware of the importance of accurate placement, as well as the various factors that could possibly contribute to poor placement.

The overall accuracy of placement of simulated ICDs in the BTS triangle of safety was low. These low accuracy rates are comparable to those found in related literature. A United Kingdom-based study assessing 50 junior doctors found that only 44% accurately located the triangle of safety, while another clinical audit showed 55% of doctors chose ICD placement inside the BTS triangle of safety [15,16].

ICD placement in the ED is common and placement outside of the accepted ‘safe’ areas is associated with higher complication rates [1,10,11,13]. An SA study by Sritharen et al. noted an overall complication rate of 16% and a meta-analysis by Hernandez et al. found a complication rate of 19% for the insertion of ICDs in trauma patients [8,17]. These significant complication rates as well as those described in other studies emphasise the importance of accurate, safe placement of ICDs [2,7,8,18]. This highlights an urgent need for intervention to improve accuracy of placement and potentially reduce complication rates. However, the rate of accuracy is not akin to the complication rate, as complications associated with depth, ectopic insertion, infection, and angle of insertion can still occur with accurate placement [5,12,18,19]. An institutional audit of placed ICDs would be required to assess complication rates, which could then be compared to accuracy of placement.

In contrast to other studies that found a significantly higher complication rate associated with ICDs placed by junior staff, this study found no difference when comparing participant rank [7,8,13,15]. This discrepancy may be a result of variations in training and clinical experience. A study that assessed anatomical placement of ICDs by Kong et al. found that only 28% of reviewed ICD placements by junior doctors (PGY-1 or PGY-2) were located within the BTS triangle of safety [8]. The authors found a significant difference when comparing PGY-1 or PGY-2 doctors as well as those who had previously attended an ATLS course [13]. These findings likely reflect improvement in accuracy with experience, which may be reflected in the results from the current study. Self-reported level of experience (number of ICDs placed) may serve as a better proxy than rank, as shown in Table 2. An audit from the United Kingdom also showed previous experience in ICD placement to be a critical factor in choosing the correct placement site [15]. The best approach to improving accuracy would thus likely be a focus on methods that build upon experience, such as simulation training, improved oversight of placement during junior years and attendance at a trauma course (e.g ATLS) [13,20].

Anatomical factors such as large body habitus have been reported to be associated with more difficult ICD placement and higher complication rates [1,19]. However, Sethuraman et al. found no significant increase in complications in patients with a large body habitus who had ICDs placed [20]. The results of the current study were not in keeping with the available literature as no statistical difference was found when comparing accurate placement according to patient BMI, although a significant difference was found when assessing placement within the BTS triangle of safety. The accuracy was, however, better in the simulated patient with the higher BMI. This outcome may have been influenced by a possible awareness by the participants of the comparison between low and high BMI as both simulated patients were in the same room. This Hawthorne effect may have resulted in participants taking greater care when placing the sticker in the simulated patient with the higher BMI [21].

The finding of a difference in accuracy and placement in the triangle of safety depending on the side of placement in the patient is a finding not in keeping with other literature either. Carter et al. found no difference when comparing left versus right sides [22]. The significance of this is uncertain. The handedness of participants and the fact that sticker placement is not as complex a motor task as actual ICD placement are possible factors which were not evaluated. This difference would be better assessed by auditing actual ICD insertions.

Placements overlying diaphragmatic structures would have significant potential for patient harm and therefore major cost implications in an already resource-constrained setting [2,9]. Avoiding such placement should thus be a particular focus during ICD insertion training. In this study, although it was found that junior doctors were significantly more likely to place the stickers over marked structures, it was more common over muscular and not diaphragmatic structures. That said, placement through large muscles should also be avoided and awareness of these anatomical structures when placing ICDs should be emphasised. The incorporation of ultrasound by junior doctors as an adjunct to locate the correct position for ICD insertion pre-procedure may ameliorate this [14].

Diaphragmatic excursion has been noted in other studies to be as low as the 7^th^ intercostal space where the diaphragm contacts the costal portion of the parietal pleura in expiration [23]. Thus, peritoneal placement becomes more likely in or below the 7^th^ intercostal space. Kwiatt et el. describe diaphragmatic excursion as rising to as high as the 4^th^ intercostal space in full expiration, thus placement lower than the 5^th^ intercostal space would be a higher risk of subdiaphragmatic placement. This is especially true in pregnant women with a gravid uterus, obese patients and patients with intraabdominal tumours or ascites [1]. In one cadaveric study it was found that more than 80% of the ICDs were placed in the 6^th^ intercostal space or below [12]. Carter et al. assessed marker placement by ED residents and consultants and found that 36.2% of placements were in the 4^th^/5^th^ intercostal space [22]. Both subgroups would be classed as senior doctors as per the current study's criteria. A much higher rate of senior doctors placed the marker in the 5^th^ intercostal space or above. Doctors with more ICD insertion experience were also more likely to place the ICD in the 5^th^ intercostal space or above. This discrepancy between findings may be a function of experience as many ICDs are placed in SA related to the high trauma and pulmonary disease caseload [2,22].

Doctors should be aware of factors that may hinder accurate ICD insertion such as female biological sex, high BMI and previous trauma or distorted anatomy [1]. These are the cases where ultrasound-guided ICD placement could be used, or alternatively, senior supervision could be requested [1,14]. A study comparing accuracy of placement within the 5^th^ intercostal space using traditional palpation techniques versus using ultrasound found that only 48% of the attempts by EM residents and students were in the correct intercostal space when using palpation. After a short hands-on training session in the use of ultrasound to identify the intercostal space, the accuracy of placement improved to 91% [14]. Ultrasound-guided placement in non-emergent cases, especially where factors have been identified that may make the insertion of an ICD more difficult, should be considered.

Departments that are responsible for many ICD placements may also consider a checklist [1]. Checklists could assist junior and inexperienced doctors to prepare correctly for the procedure and potentially make successful placement more likely. A checklist could also help make them aware of potential difficulties and offer solutions such as adequate analgesia and safe sedation methods for the combative patient. Another potential intervention could be the implementation of simulation-based training. A study by Leger et al. showed that simulation-based training improved ICD insertion success rate in a traumatic pneumothorax model [24]. This could be explored as a method to improve upon experience without having to place ICDs in a clinical setting.

### Study limitations

This was a small study with 71 participants: a small proportion of the number of doctors working within EDs in Johannesburg. Of these, few were junior doctors as the majority of ED staffing is made up of medical officers. This was a simulation-based situation and would thus not reflect the pressures involved during actual ICD insertion in an ED. The simulation setting may have resulted in participants taking greater care when placing the simulated ICD in the simulated patient with the higher BMI as part of a Hawthorne effect [21]. This study included three large academic hospitals in Johannesburg, as such there was no assessment of smaller facilities, rural facilities, or facilities in other parts of SA. Two of the centres assessed were medical emergency units which are separate departments from the trauma units. The non-trauma EDs receive fewer trauma patients, which constitutes a large proportion of patients who require ICD insertion in South Africa [2]. Doctors in those departments were less likely to be as experienced in inserting ICDs than those doctors from the trauma units. Due to anonymity, this could not be analysed separately. ICD placement was also assessed using only male simulated patients. ICD insertion is known to potentially be more difficult in female patients [1].

## Conclusion

Accurate placement of ICDs is imperative to reduce the rate of complications associated with ICD insertion. The overall accuracy of placement was worryingly low in this simulation-based study. The clinical experience of the inserting doctor was found to be a significant factor affecting the accuracy of ICD placement. Experience was found to be a common theme throughout this study and the associated literature. Methods to improve upon experience and thus accuracy should be further studied, and potentially include simulation-based training and ultrasound-guided placement. Training should emphasise the importance of recognising factors predicting difficult ICD insertion. Senior staff should be encouraged to oversee junior staff ICD placement so that immediate corrective action can be undertaken to prevent patient harm.

## Dissemination of results

Results from this study were shared with the Division of Emergency Medicine of the University of the Witwatersrand. The study and its findings may be presented at local, national or international academic meetings in the future.

## Authors contribution

Authors contributed as follow to the conception or design of the work; the acquisition, analysis, or interpretation of data for the work; and drafting the work or revising it critically for important intellectual content. TK contributed 60%, LNG 30% and TBS 10%. All authors approved the version to be published and agreed to be accountable for all aspects of the work.

## Declaration of competing interest

The authors have no conflicts of interest to declare.
